# Smell, taste and trigeminal disorders in a 65‐year‐old population

**DOI:** 10.1186/s12877-021-02242-6

**Published:** 2021-05-08

**Authors:** Anne Thea Tveit Sødal, Preet Bano Singh, Rasa Skudutyte-Rysstad, My Tien Diep, Lene Hystad Hove

**Affiliations:** 1 Department of Cariology and Gerodontology, Faculty of Dentistry, University of Oslo, P.O. Box 1109, Blindern, N-0317 Oslo, Norway; 2 Department of Oral Surgery and Oral Medicine, Faculty of Dentistry, University of Oslo, Oslo, Norway

**Keywords:** Olfaction, Gustation, Chemosensory dysfunction, Oral burning sensation, Epidemiology

## Abstract

**Background:**

Smell, taste and trigeminal disorders likely have a substantial impact on human daily life. However, data regarding the prevalence of these disorders in Norway are scarce. The aim of this study was to investigate the prevalence of smell, taste, trigeminal disorders and associated factors in a 65-year-old population in Oslo, Norway.

**Methods:**

A random sample of 223 individuals (123 men, 100 women) participated in the study. Medical history was obtained, and unstimulated whole saliva (UWS) and stimulated whole saliva (SWS) were collected to determine salivary secretion rates. Sniffin`n Sticks and Taste Strips (Burghart Messtechnik GmbH, Wedel, Germany) were used for quantitative testing of olfactory and gustatory function. In addition, the participants’ self-reported perceptions of smell and taste, and burning mouth sensation were investigated.

**Results:**

The results showed that 34 % of the participants had reduced smell (28 % hyposmia and 6 % anosmia) and 28 % had reduced taste perception (21 % hypogeusia and 7 % ageusia). 13 % of the partcipants had a combination of smell and taste disorders. Dysgeusia was reported by 5 % and burning mouth sensation (syndrome) by 3 % of the participants. Hyposmia, hypogeusia and ageusia were significantly more prevalent among men. Significant associations were found between taste disorders and previous history of cerebral hemorrhage and heart attack, and between burning mouth sensation and gastrointestinal disorders. Disturbances in olfactory, gustatory and trigeminal function were significantly related to medication use. Ageusia and burning mouth sensation were significantly more prevalent among smokers. Except from higher prevalence of ageusia among participants with hyposalivation with respect to SWS, no significant associations were found between salivary secretion rate and chemosensory or trigeminal disorders in the present study.

**Conclusions:**

The present study revealed that one-third of 65-year-old individuals had impaired smell and more than one-fourth had impaired taste function. The prevalence of dysgeusia and burning mouth sensation was very low. Reduced smell and taste perception were more common among men than women. Furthermore, some diseases and medications were associated with chemosensory and trigeminal disorders. Ageusia was associated with SWS hyposalivation.

## Background

Olfactory, gustatory and trigeminal functions are important in many aspects of human daily life. Disturbances in olfactory and gustatory function may result in reduced ability to detect smoke, e.g. fire, or other dangerous situations, poor perception of detecting one’s own body odor, detecting spoiled food and difficulties with cooking and decreased appetite [1, 2]. Smell and taste disorders may therefore affect general health and social function of individuals [3, 4]. Disturbances in trigeminal function may lead to oral burning sensation [5, 6]. In addition, trigeminal nerve endings located in the oral and nasal cavity plays an important role in detecting temperature, consistency and pungency of food and beverages [7, 8], and thereby contribute in flavor perception. Chemosensory disorders and burning mouth sensation have been reported to have a negative association with quality of life and social function [3, 9, 10]. A study investigating causes and consequences of chemosensory disorders showed that the reduction in smell and taste affected their socializing with respect to dining and ability to smell other people’s body odor [3]. Similarly, a survey among individuals suffering from olfactory disorders in a British population revealed a significant impact on both physical, social, psychological and emotional aspects [11]. The participants also complained about the lack of information and support from health care workers in coping with their condition [11]. Chemosensory disorders may also lead to an unhealthy dietary composition and an increased intake of sugar [12], and may have a detrimental effect on both the general and oral health.

The etiology of chemosensory and trigeminal disorders is multifactorial. The most common causes for olfactory dysfunction are upper respiratory infections, head trauma and nasal and paranasal sinus disease [13]. Gustatory function may be disturbed by bad-tasting substances from oral conditions like gingivitis [14]. In addition, oral dryness and oral candida infections can make the transport of tastants to taste buds difficult, or taste buds can be damaged by local trauma [14, 15]. Burning sensation in the oral mucosa can be caused by nutritional deficiency, trigeminal neuralgia, autoimmune disorders, medication, viral infection, trauma following dental treatment, among other factors [5]. Furthermore, during the Covid-19 pandemic there has been revealed increasing evidence of disturbances in olfactory, gustatory and trigeminal function in infected patients [16–18]. Moreover, disorders in the olfactory and gustatory system can be signs of underlying diseases like cancer, Alzheimer’s disease, Parkinson’s disease or diabetes [13, 14, 19]. Modifications in the grey matter distribution in the gustatory and pain matrix can lead to disturbances in perception of these senses [20]. In addition, smoking has been suggested as a possible risk factor for chemosensory and trigeminal disorders [21–24].

Previous studies have shown that men have lower smell and taste sensitivity than women [25, 26]. However, burning mouth complaints have been reported more frequently in women, especially after menopause [27–29]. Furthermore, olfactory and gustatory function have been shown to decrease with age [25, 30–33]. The reason for this may be structural changes in the oral/nasal epithelium (metaplasia) and other parts of the sensory system [34, 35] due to cumulative damage caused by harmful environmental substances and infections throughout life, combined with reduced ability to regenerate damaged cells [36, 37]. In addition, some medications may affect olfactory, gustatory and trigeminal function [3, 28, 29, 38]. Increased burden of diseases and increased medication use in elderly people, in addition to physiological age-related changes, may therefore lead to disturbed chemosensory and trigeminal function.

Epidemiological studies have shown that more than 50 % of the U.S. population older than 65 years are affected by olfactory disorders [13, 39, 40]. In a German study, gustatory and olfactory disorders were found in more than 20 % in the age group 65–74 years [23]. Tammiala-Salonen et al. found that 15 % of a Finnish adult population had experienced prolonged burning sensation in the mouth [28]. Along with the ongoing growth in the proportion of older adults in the population [41], the number of individuals with chemosensory and trigeminal disorders may increase in the years to come. Detection, diagnostics and treatment of chemosensory and trigeminal disorders is not common practice in the Norwegian health sector, and little is known about prevalence of smell, taste and trigeminal disorders in the general senior population in Norway.

Therefore, the aim of the present study was to describe the prevalence of smell, taste and trigeminal disorders in a general 65-year-old population in Oslo, Norway, and to investigate associations between these disorders and gender, smoking, salivary secretion, chronic diseases and use of medications.

## Methods

### Study design

 This cross-sectional study was part of a larger epidemiological study investigating oral health in a 65-year-old population in Oslo, Norway (The OM65-study). The main study included examinations of oral dryness [42], dental caries, endodontic and periodontal conditions among other parameters. The study was approved by the Norwegian Regional Committee for Medical and Health Research Ethics (REK 2018/1383) and performed in compliance with the tenets of the Declaration of Helsinki. All participants signed a written informed consent.

### Participants

A random sample of Oslo residents, born in 1954, was drawn from the Norwegian tax register and invitation letters were sent out. All individuals who received the letter and were reachable by phone were contacted and given the opportunity to participate in the study. The calculated sample size for the OM65-study was 450 participants. A subsample of 225 of the OM65-study participants was randomly assigned for chemosensory and trigeminal examinations. Participants were instructed not to eat, drink, use chewing gum or smoke for one hour before the examination. Data collection took place at the Research Clinic at the Institute of Clinical Dentistry, University of Oslo, from February to December 2019.

### Questionnaire

Participants answered a semi structured, self-administered questionnaire, distributed by email using an internet link to the Nettskjema software (University of Oslo, Norway) prior to the clinical examination. The questionnaire contained items regarding the participants’ gender, general health, medication use and smoking habits. Participants’ self-reported health status was assessed and included diseases and medications presented in Table 1. The question assessing smoking status had three response alternatives: never smoker, former smoker and current smoker. Current smoker was defined as individuals who smoke ≥ 1 cigarette daily.

### Saliva assessment

 Unstimulated whole saliva (UWS) and stimulated whole saliva (SWS) were collected from all participants. Both UWS and SWS was collected for 5 min. Before the collection of UWS started, participants were instructed to swallow any saliva in their mouth and then spit into a pre-weighed cup when needed and also to avoid swallowing during the collection time. For SWS measurements, the participants first chewed on a paraffin tablet (Ivoclar Vivadent, Schaan, Lichtenstein) for 30 s, swallowed any saliva in their mouth, and then continued chewing and when needed spat saliva into a pre-weighed cup for 5 min. The cup was chilled on ice before and during the collection time. After collection of saliva the cup was weighed and secretion rate calculated as ml/min (1 g/min = 1 ml/min). Hyposalivation was defined as a secretion rate of ≤ 0.1 ml/min for UWS and ≤ 0.7 ml/min for SWS [43].

### Assessment of dysgeusia and burning mouth sensation

 The participants were interviewed regarding their experience of dysgeusia and burning mouth sensation using questions prepared by Dr. P.B. Singh and validated at the Dry Mouth Clinic at Faculty of Dentistry, University of Oslo [44]. The interview contained both binary, multiple choice and open-ended questions.

### Olfactory assessment

 Prior to the olfactory testing participants were asked to score their smell perception on a linear visual analogue scale (VAS) from 0 to 10, where 0 = no smell perception and 10 = very good smell perception. An identification method, Sniffin` Sticks-Screening test (Burghart Messtechnik GmbH, Wedel, Germany) consisting of 12 felt-tip odor pens was used for non-lateralized psychophysical testing of olfactory function [30, 45, 46]. The participants were informed about the procedure before the test started. Each pen was placed approximately 3 cm from both nostrils for 3–4 s. Then, the participants were instructed to choose one alternative from a multiple-choice card with four odor alternatives using a forced-choice procedure. The answers were recorded as 1 = correct or 0 = incorrect, and summarized (score range 0–12). A normative classification described by Hummel et al. [47] was used to categorize participants into anosmic (score 0–5), hyposmic (score 6–9) and normosmic (score 10–12).

### Gustatory assessment

Prior to the gustatory testing participants were asked to score their taste perception on a linear visual analogue scale (VAS) from 0 to 10, where 0 = no taste perception and 10 = very good taste perception. Gustatory function was measured by Taste Strips (Burghart Messtechnik GmbH, Wedel, Germany) impregnated with solutions in four different concentrations of four different taste qualities; sweet (0.4, 0.2, 0.1, 0.05 g/mL sucrose), sour (0.3, 0.165, 0.09, 0.05 g/mL citric acid), salty (0.25, 0.1, 0.04, 0.016 g/mL sodium chloride) and bitter (0.006, 0.0024, 0.0009, 0.0004 g/mL quinine-hydrochloride). The whole mouth taste test was performed by the taste strip first being placed on the anterior tip of the tongue. Then the participant was instructed to close his/her mouth and rub the taste strip back and forth. The participants were instructed to choose between the four taste qualities or no taste after tasting each taste strip [48]. The responses were recorded as 1 = correct or 0 = incorrect, and summarized (score range 0–16). The taste strips were administered in the same order for each participant from the lowest stimulus amount (concentration) for all taste qualities to the highest. Before starting the test, the participants tasted a taste strip with no taste, and between each taste strip, the participants rinsed their mouth with water. Participants were classified into ageusic (score 0–4), hypogeusic (score 5–8) and normogeusic (score 9–16) by a normative classification as described by Landis et al. [25].

### Statistical analyses

Data from the clinical examinations were collected in The Oral Data Collector, a datasheet designed for this study using Microsoft Excel 2016 (Microsoft Corporation, Redmond, Washington, US), and imported into STATA (Stata version 16.1; College Station, TX, USA) for statistical analysis. Data were stored in Service for Sensitive Data (TSD facilities, UiO). The results from the descriptive analyses are presented as percentage distributions or median and interquartile range (IQR). Chi-square or Fischer’s exact test were used to compare categorical variables. Non-parametric tests (Mann-Whitney U test or Kruskal-Wallis ANOVA) were used to detect median differences between the groups of continuous variables. All differences were considered statistically significant at *p* < 0.05.

## Results

### Background information

Of the 797 eligible individuals who were reached by phone, 460 individuals accepted the invitation to participate in the OM65 study (response rate 58 %). Of the 225 participants who were randomly assigned for the chemosensory examinations and interview, one participant who did not complete the questionnaire and one participant who failed to complete the olfactory and gustatory test due to discomfort were excluded from the analyses. The distribution of participants in relation to background characteristics is presented in Table 1.

**Table 1:** Background characteristics of participants in the study

Participant characteristics	% (n)
	(*N* = 223)
**Gender**	
Male	55 (123)
Female	45 (100)
**Smoking**	
Never smoker	41 (91)
Former smoker	48 (106)
Current smoker	12 (26)
**Diseases**	
Cerebral hemorrhage	4 (8)
Heart attack	6 (14)
Gastrointestinal disease	2 (5)
**Medication type**	
Antidepressants	4 (8)
Anticoagulants	21 (46)
Antacid medication	9 (19)
Asthma medication	6 (14)
Corticosteroids^a^	2 (4)
Hormone medication^b^	13 (28)
**Hyposalivation**	
Stimulated saliva	5 (12)
Unstimulated saliva	10 (22)

^a^exc. asthma and allergy medication, ^b^incl. thyroid hormones

No participants reported antibiotic use

### Olfactory function

**Fig. 1:**
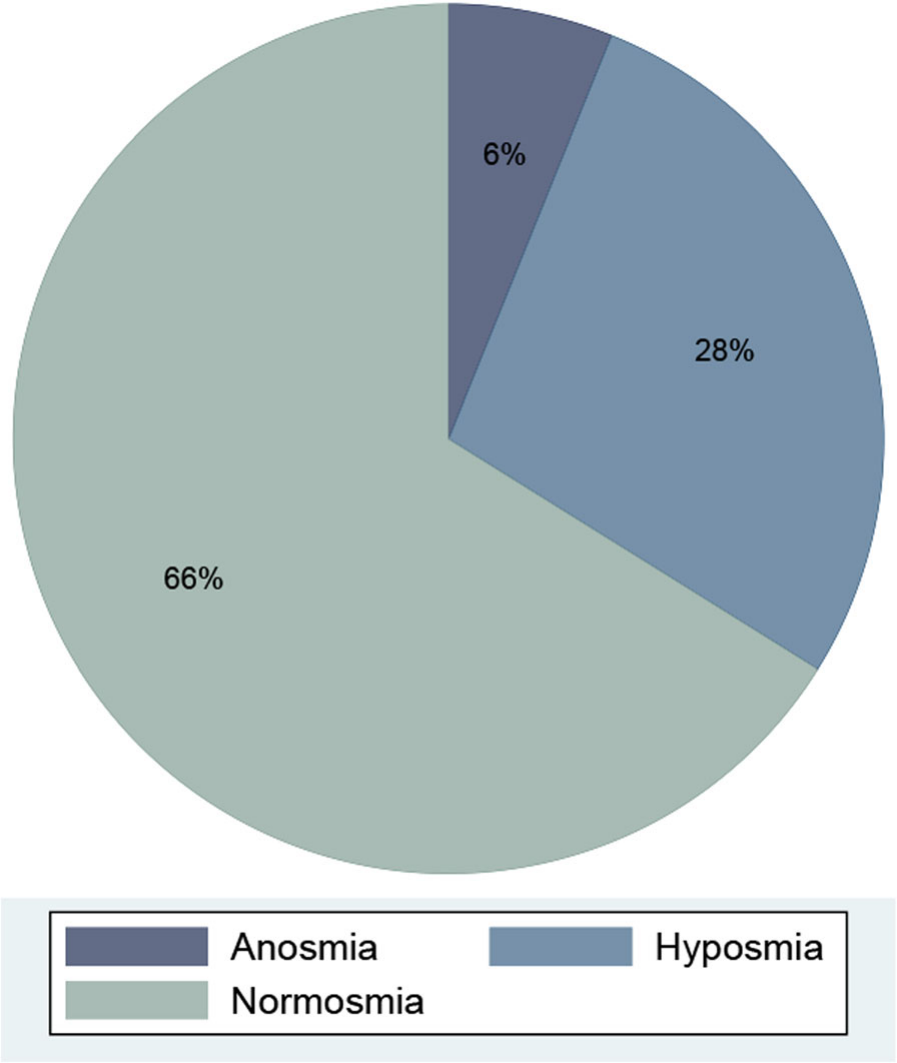
Percentage distribution of participants with normosmia, hyposmia and anosmia by Sniffin Sticks-Screening test. *N* = 223

The prevalence of normosmia, hyposmia and anosmia according to the Sniffin` Sticks-Screening test is presented in Fig. 1. The results showed that 34 % of participants had reduced olfactory function. Fourteen participants identified only 5 or less of the 12 odors and were classified as functionally anosmic. Sixty-two participants recognized between 6 and 9 of the 12 odors and were classified as hyposmic.

**Fig. 2:**
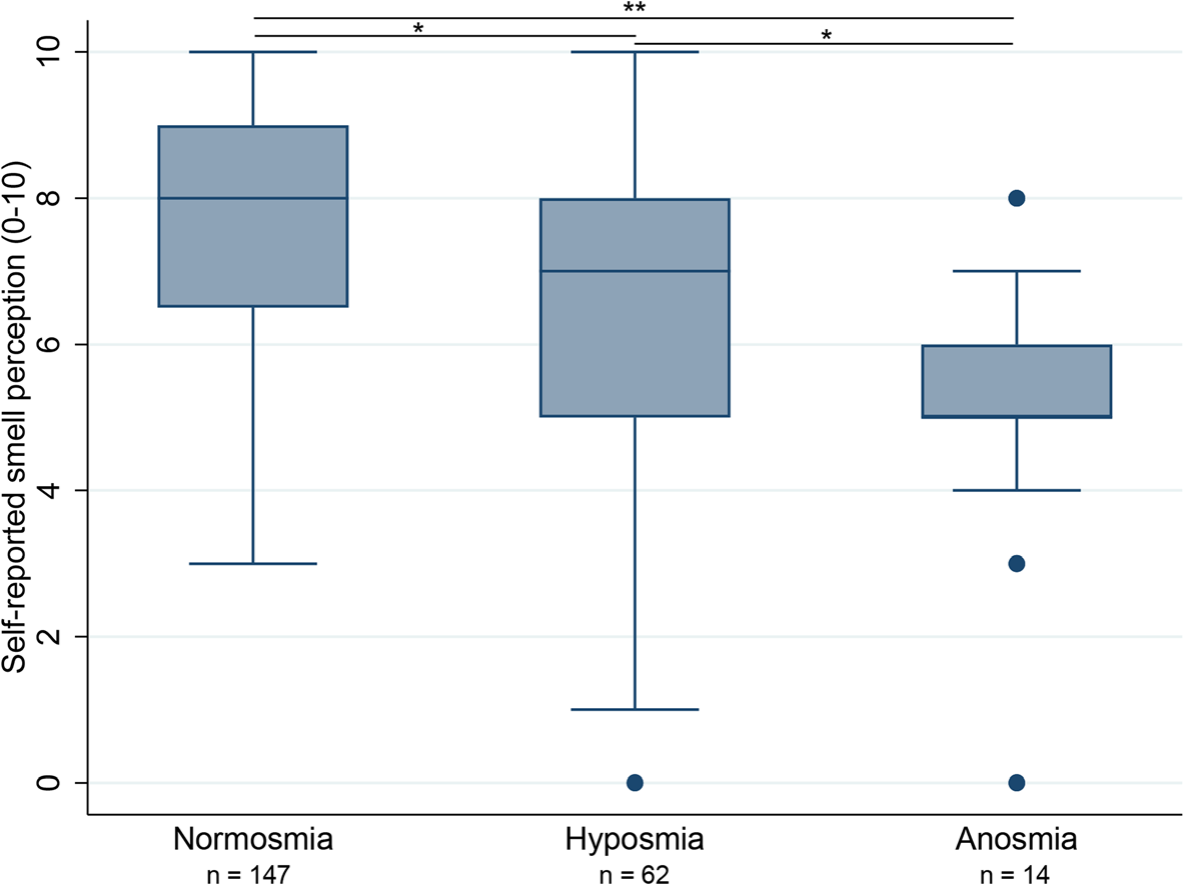
Individual self-reported smell perception scores (VAS) in normosmic, hyposmic and anosmic participants (Sniffin` Sticks-Screening test). *N* = 223. Boxplots illustrating medians with interquartile ranges (IQRs) of self-reported smell perception (VAS; 0–10) in normosmic, hyposmic and anosmic participants. Kruskal-Wallis, Mann-Witney U test; *p < 0.05, ** *p* < 0.001. Dots in the figure represent outliers

Results from the self-reported smell identification assessment showed a median VAS-score of 7 (IQR 6–8). VAS scores were associated with results from Sniffin` Sticks-Screening test (Fig. 2). Individuals classified as normosmic had a significantly higher median VAS-score (median 8.0, IQR 6.5-9.0) than those classified as hyposmic (median 7.0, IQR 5.0–8.0; *p* = 0.003) and anosmic (median 5.0, IQR 5.0–6.0; *p* < 0.001). The median VAS-score for participants classified as hyposmic were significantly higher than for participants classified as anosmic (*p* = 0.039).

### Gustatory function

**Fig. 3:**
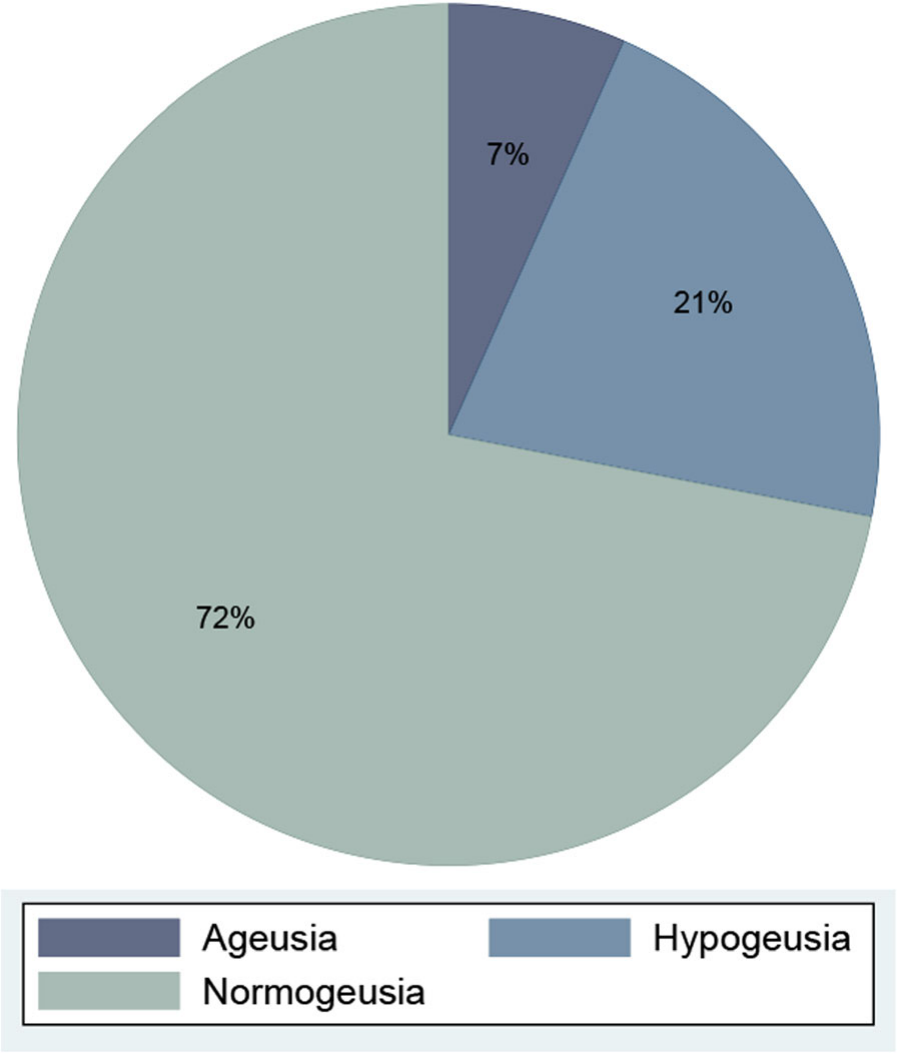
Percentage distribution of participants with normogeusia, hypogeusia and ageusia by Taste Strips test. *N* = 223

**Fig. 4:**
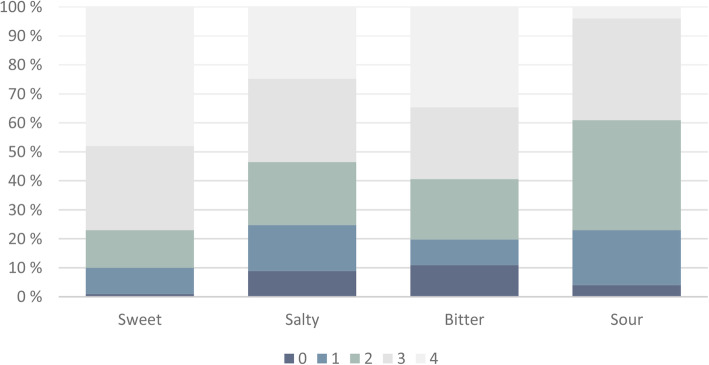
Percentage distribution of participants by total score (0–4) in the four taste qualities. *N* = 223

The distribution of participants in relation to gustatory test score by taste strips is illustrated in Fig. 3. The results showed that more than one fourth of the participants had reduced gustatory function. Fifteen participants had a total score of 4 or less and were classified as functionally ageusic. Forty-seven participants had a total score between 5 and 8 and were classified as hypogeusic. Sweet taste was most frequently identified correctly in all four concentrations, while sour taste was least frequently identified correctly in all four concentrations (Fig. 4). Bitter taste was least often identified correctly in at least one of the concentrations (Fig. 4). Median self-reported taste perception score (VAS) was 7 (IQR 6–8). No significant differences in VAS-scores were found between participants classified as normogeusic (median 7, IQR 6–8), hypogeusic (median 7, IQR 6–8) and ageusic (median 7, IQR 5–8).

Twelve participants (5 %) reported dysgeusia, and answered further questions regarding frequency of dysgeusia and taste characteristic. The frequency of dysgeusia was reported as constant (1 case), daily (3 cases), sometimes (7 cases) or only in bad periods (1 case). One dysgeusic participant did not report the frequency of dysgeusia. Metallic taste dysgeusia was the most common complaint and reported by 5 of the dysgeusic participants. Other taste dysgeusias reported were bitter (1 case), rotten (2 cases) and harsh (2 cases). Two participants who reported dysgeusia did not specify the taste.

### Burning mouth sensation

Eight participants (4 %) reported that they had experienced burning mouth sensation. The burning sensation was located to the entire tongue (3 cases), the anterior part of the tongue (1 case), the side of the tongue (1 case), the gingiva (1 case) or the palate and the gingiva (1 case). One of the participants experiencing burning mouth sensation did not specify the location.

### Combinations

**Fig. 5:**
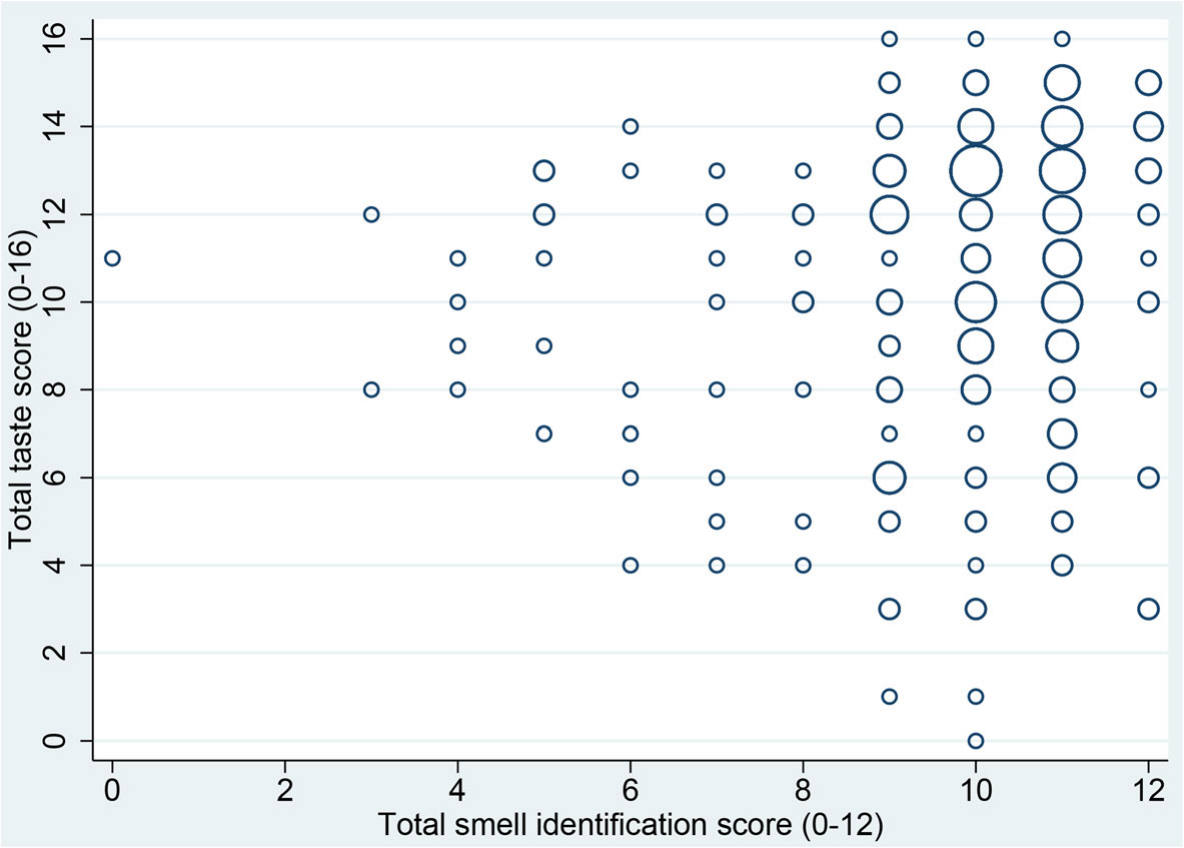
Scatter plot showing smell (Sniffin Sticks’) and taste (Taste Strips) scores combined for all participants. *N* = 223. The smallest circles represent 1 observation. Larger circles represents higher numbers of identical observations

Smell and taste scores combined for all participants are shown in Fig. 5. Twenty-eight participants (13 %) had a combination of a smell disorder (hyposmia or anosmia) and a taste disorder (hypogeusia or ageusia). Burning mouth sensation was accompanied by a quantitative smell or taste disorder in 6 cases (3 %). Three participants (1 %) reported a combination of qualitative (dysgeusia) and quantitative taste disorder (hypogeusia/ageusia). Six participants (3 %) both reported a combination of qualitative taste disorder (dysgeusia) and had quantitative smell disorder (hyposmia/anosmia). Eleven participants of those with anosmia (79 %) had normal taste function. Nine participants of those with ageusia (60 %) had normal smell perception.

### Factors associated with olfactory and gustatory dysfunction and burning mouth sensation

#### Olfactory dysfunction

**Fig. 6:**
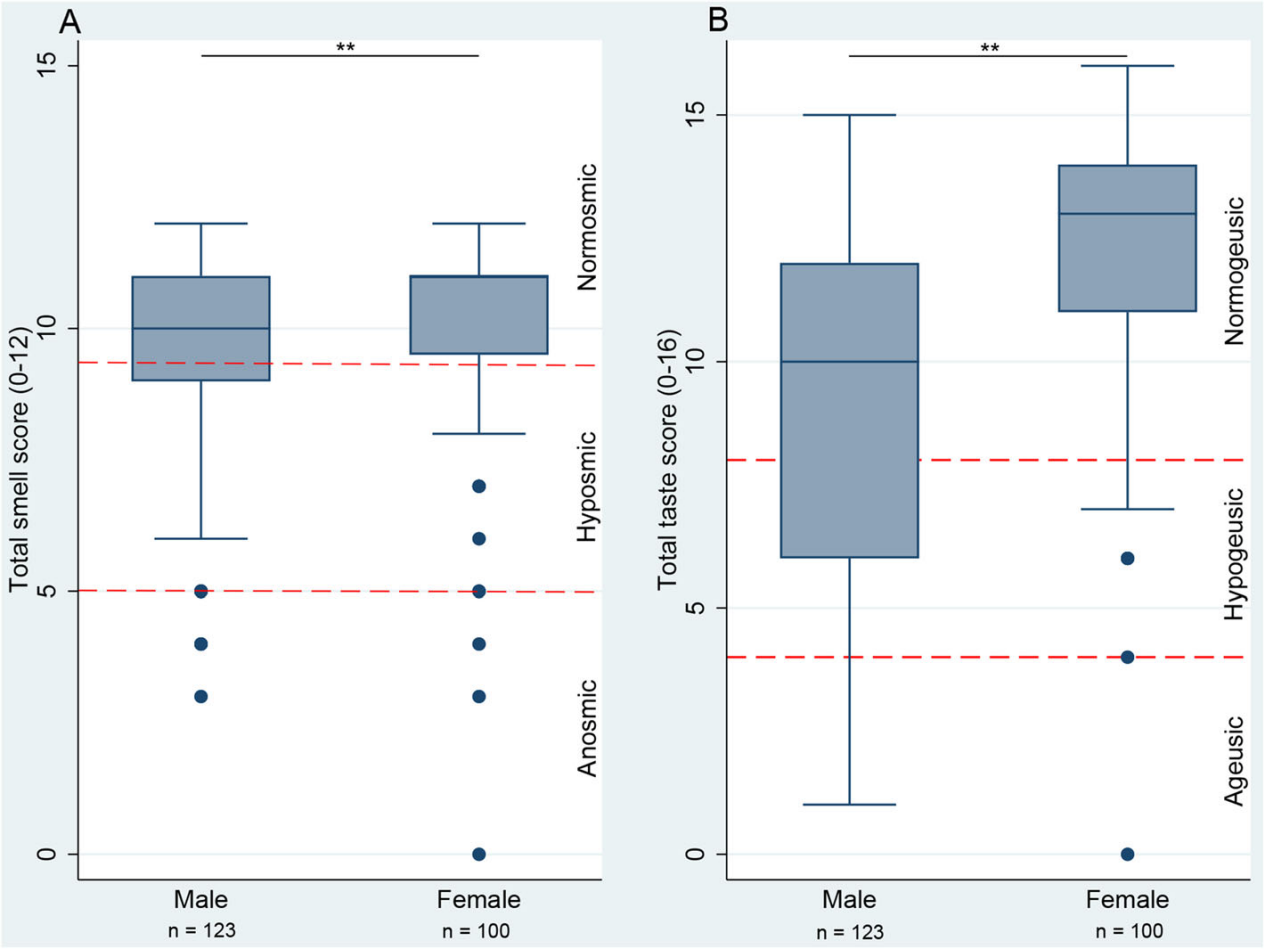
Gender differences in olfactory (**a**) and gustatory (**b**) test scores. *N* = 223. Boxplots illustrate medians with interquartile ranges (IQRs) of measured smell score (**a**) and taste score (**b**) in males and females. Mann-Whitney U test; ** *p* < 0.001. Dots in the figure represent outliers. Dashed red lines represent score limit for hyposmic/hypogeusic (upper) and score limit for anosmic/ageusic (lower)

According to Sniffin` Sticks-Screening test women had significantly higher median total smell score (median 11, IQR 9.5–11) than men (median 10, IQR 9–11) (*p* = 0.002) (Fig. 6a). A significantly greater proportion of men (34 %) than women (20 %) was classified as hyposmic according to olfactory test score (*p* < 0.001).

No significant association was found between reduced olfactory function and smoking or salivary secretion rates. Associations between olfactory function and diseases and medication use are shown in Table 2. Anosmia was significantly more prevalent among participants using corticosteroids (except asthma and allergy medication) than those who did not (*p* = 0.020). No significant associations were found between hyposmia and diseases or medication use.

#### Gustatory dysfunction

According to the taste strips test, women had significantly higher median total taste score (median 13, IQR 11–14) than men (median 10, IQR 6–12) (*p* < 0.001) (Fig. 6b). A significantly greater proportion of men than women was classified as hypogeusic (33 % vs. 7 %; *p* < 0.001) and ageusic (11 % vs. 2 %; *p* = 0.014) according to the gustatory test score.

The prevalence of ageusia was significantly higher in current smokers (19 %) than in former smokers (6 %; *p* = 0.025) and never smokers (4 %; *p* = 0.025). A significantly higher prevalence of ageusia was found among participants who had hyposalivation with respect to SWS (25 %) compared to participants without hyposalivation with respect to SWS (6 %) (*p* = 0.038). No significant association was found between gustatory function and UWS secretion rate. Table 2 shows associations between gustatory function and diseases and medication use. Ageusia was significantly more prevalent among those who reported previous heart attack (*p* = 0.009). A significantly higher proportion of those who used antidepressants (*p* = 0.001) and blood thinners (*p* = 0.018) were classified as ageusic. Hypogeusia was significantly more common among participants who reported a history of cerebral hemorrhage (*p* = 0.012) and those who did not use hormone medication (*p* = 0.012) compared with their counterparts.

#### Burning mouth sensation

There was a tendency of higher prevalence of burning mouth sensation among women (6 %) than men (2 %), however, the difference was not statistically significant. Burning mouth sensation was significantly more prevalent in current smokers (8 %; *p* = 0.048) and former smokers (7 %; *p* = 0.032) than never smokers (0 %).

No significant association was found between salivary secretion rates and burning mouth sensation. A significantly higher proportion of those who used antacid (*p* = 0.002) and asthma medication (*p* = 0.009) complained about burning mouth sensation. Burning mouth sensation was more prevalent among those who suffered from gastrointestinal diseases (*p* = 0.011).

**Table 2:** Associations between chemosensory disorders, dysgeusia and burning mouth sensation (BMS) and diseases and medication use. *N* = 223

	Hyposmia	Anosmia	Hypogeusia	Ageusia	Dysgeusia	BMS
	**% (n)**	**% (n)**	**% (n)**	**% (n)**	**% (n)**	**% (n)**
**Cerebral hemorrhage**						
Yes (*n* = 8)	50 (4)	0 (0)	**63 (5)**	25 (2)	13 (1)	0 (0)
No (*n* = 215)	27 (58)	7 (14)	**20 (42)**	6 (13)	5 (11)	4 (8)
**Heart attack**						
Yes (*n* = 14)	29 (4)	0 (0)	14 (2)	**29 (4)**	0 (0)	0 (0)
No (*n* = 209)	28 (58)	7 (14)	22 (45)	**5 (11)**	6 (12)	4 (8)
**Gastrointestinal disease**						
Yes (*n* = 5)	20 (1)	0 (0)	20 (1)	0 (0)	20 (1)	**40 (2)**
No (*n* = 218)	28 (61)	6 (14)	21 (46)	7 (15)	5 (11)	**3 (6)**
**Antidepressant**						
Yes (*n* = 8)	38 (3)	13 (1)	13 (1)	**38 (3)**	13 (1)	0 (0)
No (*n* = 215)	27 (59)	6 (13)	21 (46)	**6 (12)**	5 (11)	4 (8)
**Blood thinners**						
Yes (*n* = 46)	28 (13)	7 (3)	30 (14)	**15 (7)**	7 (3)	4 (2)
No (*n* = 177)	28 (49)	6 (11)	19 (33)	**5 (8)**	5 (9)	3 (6)
**Antacid**						
Yes (*n* = 19)	37 (7)	11 (2)	37 (7)	0 (0)	16 (3)	**21 (4)**
No (*n* = 204)	27 (55)	6 (12)	20 (40)	7 (15)	4 (9)	**2 (4)**
**Asthma medicine**						
Yes (*n* = 14)	29 (4)	7 (1)	21 (3)	0 (0)	14 (2)	**21 (3)**
No (*n* = 209)	28 (58)	6 (13)	21 (44)	7 (15)	5 (10)	**2 (5)**
**Corticosteroids**^a^						
Yes (*n* = 4)	25 (1)	**50 (2)**	25 (1)	0 (0)	25 (1)	0 (0)
No (*n* = 219)	28 (61)	**6 (12)**	21 (46)	7 (15)	5 (11)	4 (8)
**Hormone medication**^b^						
Yes (*n* = 28)	14 (4)	4 (1)	**4 (1)**	0 (0)	11 (3)	0 (0)
No (*n* = 195)	30 (58)	7 (13)	**24 (46)**	8 (15)	5 (9)	4 (8)

Chi-square or Fisher’s exact test. *p* < 0.05 is marked with bold text

^a^Corticosterioids exc. asthma and allergy medication

^b^Hormone medication incl. thyroid hormones

## Discussion

The present study provides comprehensive data regarding prevalence of smell, taste and trigeminal disorders and associated factors in a 65-year-old population in Oslo. To our knowledge, these data and comparison of all three conditions in a general population are limited. This study revealed that olfactory and gustatory disorders are common conditions in this age group of the general population.

One third of the participants had smell disorders and more than one fourth had taste disorders. Our findings are in accordance with a study by Vennemann et al. who found the same prevalence of anosmia (6 %), but a slightly lower prevalence of hyposmia (20 %) in the age group 65–74 years in a German population [23]. In a study by Brämerson et al. of a Swedish population of adults 20 years and older, 13 % of the participants had hyposmia and 6 % had anosmia, and a significant negative correlation was found between reduced olfactory function and increasing age [32]. Other studies have also reported a decrease in olfactory function related to aging [31, 39, 49, 50]. Similar results have been found in studies where other smell identification tests were used [51].

Regarding taste disorders, a substantially higher prevalence was found than reported in the literature, ranging from 3 to 20 % [23, 31, 38, 52]. Similar as for smell disorders, a decrease in taste function related to aging has been reported [31, 52–54]. The present study only included 65-year-old individuals, which may explain the relative high prevalence compared to studies including younger age groups.

In the present study, sweet taste was most frequently, while sour taste was least frequently identified accurately in all four concentrations. This is in accordance with previous studies showing that elderly individuals’ ability to identify bitter, sour and salt taste is more commonly reduced than the ability to identify sweet taste [31, 54]. Although the association between taste ability, taste preferences and food choices is not fully understood [55], it is important to recognize that changes in taste perception might affect individuals’ dietary choices and nutritional status, which would likely be detrimental for both the general and oral health. The ability to identify umami taste was not tested in the present study because the standardized taste test kit used did not include umami strips.

The prevalence of burning mouth sensation in the present study was low and within previously reported prevalence data ranging from below 1–15 % [22, 28, 29]. Burning mouth sensation has been referred to under several names in the literature, i.e. burning mouth syndrome, burning mouth, glossodynia, glossopyrosis [5], and the varying prevalences may be due to different diagnostic criteria used in different studies.

In the present study, women showed an overall increase in smell and taste perception when compared to men. This finding is consistent with previous literature [23, 31, 32, 50], however, the mechanisms for gender differences in chemosensory perception are not fully understood. It might be speculated that hormonal differences, structure and physiology of the sensory organs as well as training of the chemosensory functions may affect smell and taste perception, but this needs to be investigated further.

The prevalence of ageusia was significantly higher in current smokers than in former and never smokers. The effect of smoking on olfactory and gustatory function in previous literature is not consistent. Some studies have shown an association between being smoker and reduced olfactory and gustatory function [23, 24, 50, 56], while others did not [32, 49, 57]. Furthermore, burning mouth sensation in the present study was significantly more prevalent in “current smokers” and “former smokers” than “never smokers”, which is in consistency with previous literature [21].

Saliva has been described as an important factor for solubilization and transport of tastants, as well as maintenance of taste buds [15]. Except for higher prevalence of ageusia among individuals with hyposalivation with respect to SWS compared to those with normal SWS secretion rate, no other significant associations were found between salivary secretion rate and chemosensory or trigeminal disorders in the present study. Rusthen et al. found a higher prevalence of smell, taste and trigeminal disorders in patients with Sjögren´s syndrome with reduced salivary secretion rates compared to healthy controls [33], but no significant correlations were found between salivary secretion rate and chemosensory or trigeminal disorders [33]. Other studies have shown a negative correlation between salivary secretion rate and taste function [58, 59]. Previously reported data suggests that several salivary parameters have an effect on taste perception [60–62]. This might be due to differences in saliva composition, i.e. buffer capacity and amount of proteins [58], which indicates that qualitative characteristics of saliva might be important for taste function. Other salivary qualities than secretion rate were not investigated in the present study.

A number of diseases and medications have shown to be associated with disturbances in gustatory, olfactory and trigeminal function [3, 28, 38, 63, 64]. In addition, chemosensory disturbances can be early symptoms of other serious underlying conditions, i.e. cancer, neurodegenerative and neurological disorders, and metabolic and endocrine diseases [13, 14, 19], which emphasize the importance of awareness of these disorders in the general population. In the present study, higher prevalence of taste disorders was found among individuals with a history of cerebral hemorrhage and previous heart attack. Burning mouth sensation was more prevalent among individuals with gastrointestinal disorders. In addition, use of certain medication types was significantly associated with disturbances in olfactory and gustatory function and burning mouth sensation. Some antibiotics might lead to disturbances in gustatory function [63]. Antibiotic treatment may therefore result in a transient increase in the prevalence of taste disorders, however, use of antibiotics was not reported by any of the participants in the present study.

The response rate in the OM65 study was 58 %, leading to a sizable proportion of non-respondents and possibility for selection bias. The selection of individuals from the target population was random, however, several factors may have influenced whether individuals agreed to participate or was reachable by phone. Individuals with severe illness or people living in institutions may have had difficulties answering the invitation and with participation. This may have led to a healthier study population compared to the target population. When compared to statistics from the Norwegian Prescription Database [65], a lower proportion of the participants used antidepressants, anticoagulants, antacid medication, asthma medication, corticosteroids and hormone medication compared to the target population. This might indicate that the prevalence of chemosensory and trigeminal disorders in the general population can be even higher than what was found in this study.

The present study revealed several risk indicators for chemosensory and trigeminal disorders which would be interesting to study further. However, the number of participants with specific diseases or use of medications were low and several associations did not reach statistical significance. A cross-sectional study design makes it difficult to distinguish between side effects of medications and the underlying medical conditions. Furthermore, the self-reported data on general health, smoking habits and medication use may be subject to recall bias.

Due to different methods used when investigating olfactory and gustatory function, direct comparison of available studies can be challenging. For olfactory testing, some studies have included threshold, discrimination and identification tests, resulting in a TDI-score, which may give a broader picture of the olfactory function [30, 47]. An individual’s semantic ability and familiarity with the smells included in the identification test can influence the results when only the identification test is used. In addition, a complete TDI-score would be necessary to establish an age and gender specific diagnosis. However, the present study was part of a larger epidemiological study and due to time limitation only the identification test was included. In addition, familiarity with odors used in the identification test might be influenced by ethnicity. However, associations between olfactory function and ethnicity was not investigated in the present study, as 91 % of participants were Caucasian and the remaining group was too small and heterogeneous.

Hyposmic and anosmic individuals scored their own smell perception significantly lower than normosmic individuals on a linear visual analogue scale ranging from very bad (0) to very good (10). However, the median VAS-score was five or higher in both the anosmic and hyposmic group, which may indicate a low awareness of disturbance in olfactory function among affected individuals. No statistically significant differences in median VAS-score for self-reported taste perception values in normogeusic, hypogeusic and ageusic participants were found. The decrease in olfactory and gustatory function related to aging usually is a gradual process, and might therefore be habituated and lead to a reduced awareness compared to individuals who experience a sudden loss in function [66].

The results in the present study showed that smell and taste disorders are common in the general Norwegian 65-year-old population. The findings are in accordance with existing evidence showing a decrease in chemosensory function related to aging [31, 32, 49, 52]. Whether this decrease can be considered a natural aging process rather than a pathologic condition remains unknown. The majority of affected individuals had low awareness of reduced smell and taste function, which might suggest that disorders had limited impact on their daily function. On the other hand, despite the seemingly low awareness among affected individuals, it is important to highlight the prevalence of chemosensory disorders in the aging population due to the possible hidden impact on an individual`s daily life, i.e. difficulties of detecting smoke or other dangerous situations, detecting spoiled food and potential toxins or change in diet [1–4, 11]. Given the decrease in olfactory and gustatory function related to aging [31, 32, 49, 52], it can be speculated that a further deterioration and the impact of smell and taste disorders on daily function may be even more considerable in individuals older than 65 years. Further research is needed in order to establish how chemosensory disorders affect daily life and functioning of aging individuals.

## Conclusions

 In the present study one-third of the participants had impaired smell function and more than one fourth had impaired taste function. The prevalence of dysgeusia and burning mouth sensation were low. Reduced smell and taste functions were more common among men than women. Furthermore, some diseases and medications were associated with chemosensory disorders. The prevalence of ageusia was significantly higher among participants with SWS hyposalivation compared to those with normal SWS secretion rate. In addition, an overall low awareness of smell and taste disorders among affected individuals was observed.

## Data Availability

The datasets used and/or analyzed during the current study are available from the corresponding author upon reasonable request.

## Abbreviations

UWS: Unstimulated whole saliva
SWS: Stimulated whole saliva
VAS: Visual analogue scale
BMS: Burning mouth sensation
STATA: Software for statistics and data science
IQR: Interquartile range
TSD: Tjeneste for sensitive data
